# A Study of Alternative TrkA Splicing Identifies TrkAIII as a Novel Potentially Targetable Participant in PitNET Progression

**DOI:** 10.3390/biology13030171

**Published:** 2024-03-07

**Authors:** Maddalena Sbaffone, Marie-Lise Jaffrain-Rea, Lucia Cappabianca, Francesca Carbonara, Francesca Gianno, Tiziana Feola, Marianna Ruggieri, Veronica Zelli, Rita Maccarone, Stefano Guadagni, Marco Clementi, Antonietta Arcella, Vincenzo Esposito, Giulia Carozza, Ilaria Martelli, Antonietta Rosella Farina, Andrew Reay Mackay

**Affiliations:** 1 Department of Biotechnological and Applied Clinical Sciences, University of L’Aquila, Via Vetoio, 67100 L’Aquila, Italy; maddalena.sbaffone@graduate.univaq.it (M.S.); marielise.jaffrain@univaq.it (M.-L.J.-R.); luciaannamaria.cappabianca@univaq.it (L.C.); francesca.carbonara@graduate.univaq.it (F.C.); marianna.ruggieri@graduate.univaq.it (M.R.); veronica.zelli@univaq.it (V.Z.); rita.maccarone@univaq.it (R.M.); marco.clementi@univaq.it (M.C.); giulia.carozza@graduate.univaq.it (G.C.); ilaria.martelli@graduate.univaq.it (I.M.); antonietta.farina@univaq.it (A.R.F.); 2 Neuromed, Istituti di Ricovero e Cura a Carattere Scientifico (IRCCS), 86077 Pozzilli, Italy; francesca.gianno@uniroma1.it (F.G.); tiziana.feola@uniroma1.it (T.F.); arcella@neuromed.it (A.A.); vincenzo.esposito@uniroma1.it (V.E.); 3 Department of Radiological, Oncological and Pathological Sciences, La Sapienza University of Rome, 00185 Rome, Italy; 4 Department of Experimental Medicine, La Sapienza University of Rome, 00185 Rome, Italy; 5 Department of Neurology and Psychiatry, La Sapienza University of Rome, 00185 Rome, Italy

**Keywords:** PitNETs, alternative splicing, *TrkAIII* splice variant, HIF2α, splice factors SF3B1, U2AF and SRSF2, hotspot SF3B1 mutation, Xbp1, JCPyV large T antigen

## Abstract

**Simple Summary:**

Pituitary neuroendocrine tumors (PitNETs) develop from anterior pituitary cells and, although generally benign, comprise a small subset of therapy-resistant aggressive or metastatic tumors. This highlights the need to identify novel potential therapeutic targets. PitNETs have low rates of somatic mutation and their pathogenesis is poorly understood. PitNETs are associated with conditions linked to alternative splicing, which may activate oncogenic pathways, and express the neurotrophin receptor tropomyosin receptor kinase A (TrkA), which exhibits oncogenic alternative *TrkAIII* splicing in other neuroendocrine tumors. In this study, we report for the first time that alternative *TrkAIII* mRNA splicing is common in PitNETs and can associate with intracellular TrkAIII activation, identifying TrkAIII as a novel potential targetable oncogenic participant in PitNET pathogenesis and progression.

**Abstract:**

Pituitary neuroendocrine tumors (PitNETs) are generally benign but comprise an aggressive, invasive, therapy-resistant, metastatic subset, underpinning a need for novel therapeutic targets. PitNETs exhibit low mutation rates but are associated with conditions linked to alternative splicing, an alternative oncogene pathway activation mechanism. PitNETs express the neurotrophin receptor TrkA, which exhibits oncogenic alternative *TrkAIII* splicing in other neuroendocrine tumors. We, therefore, assessed whether *TrkAIII* splicing represents a potential oncogenic participant in PitNETs. *TrkAIII* splicing was RT-PCR assessed in 53 PitNETs and TrkA isoform(s) expression and activation were assessed by confocal immunofluorescence. *TrkAIII* splicing was also compared to HIF1α, HIF2α, SF3B1, SRSF2, U2AF1, and JCPyV large T antigen mRNA expression, Xbp1 splicing, and *SF3B1* mutation. *TrkAIII* splicing was detected in all invasive and most non-invasive PitNETs and was significantly elevated in invasive cases. In PitNET lineages, *TrkAIII* splicing was significantly elevated in invasive PIT1 PitNETs and high in invasive and non-invasive SF1 and TPIT lineages. Immunoreactivity consistent with TrkAIII activation characterized PitNET expressing *TrkAIII* mRNA, and invasive Pit1 PitNETs exhibited elevated *HIF2α* expression. *TrkAIII* splicing did not associate with *SF3B1* mutations, altered *SF3B1*, *SRSF2*, and *U2AF1* or JCPyV large T antigen expression, or Xbp1 splicing. Therefore, *TrkAIII* splicing is common in PitNETs, is elevated in invasive, especially PIT1 tumors, can result in intracellular TrkAIII activation, and may involve hypoxia. The data support a role for *TrkAIII* splicing in PitNET pathogenesis and progression and identify TrkAIII as a novel potential target in refractory PitNETs.

## 1. Introduction

Pituitary neuroendocrine tumors (PitNETs) originate from cells of the anterior pituitary and are classified by immunohistochemistry, according to pituitary transcription factor and hormone expression [1,2,3,4,5]. Several morpho-functional PitNET phenotypes can be identified, along with three lineages of origin. These phenotypes include the following: functioning lactotroph, somatotroph, and thyrotroph PitNETs positive for pituitary-specific transcription factor 1 (PIT1-PitNETs); corticotroph PitNETs positive for T box transcription factor (TPIT-PitNETs); gonadotroph PitNETs positive for Steroidogenic factor 1 (SF1-PitNETs); silent/non-functioning PIT1, SF1, or TPIT PitNET sub-types, pluri-hormonal PitNETs and “null cell” PitNETs [1,2]. PitNETs are classified as functioning when they are associated with bio-clinical evidence of hormone hypersecretion. 

Despite being typically benign, approximately 40% of PitNETs invade surrounding structures, and a small proportion of mainly lactotroph and corticotroph PitNETs develop into aggressive, therapy-resistant/refractory, sporadically metastatic tumors [2,6,7]. Aggressive and metastatic PitNETs share a number of bio-clinical features, despite a lack of specific molecular markers [8], and current guidelines recommend treatment with temozolomide [6,7]. However, primary and secondary therapeutic resistance to temozolomide is frequent [7], emphasizing the need to identify novel therapeutic targets for this specific subgroup, which continues to pose a significant therapeutic challenge.

PitNETs, in general, are sporadic tumors that exhibit low oncogene mutation rates, chromosomal alterations, transcriptomic, and epigenetic signatures [5,9,10,11]. However, they do associate with several conditions that have been linked to alternative splicing, including hypoxia [12], oxidative stress [13], somatic mutations in the splicing factor 3b subunit 1 (*SF3B1*) [14,15,16], and dysregulated splicing machinery [17]. PitNETs have also been linked to the neurotropic John Cunningham polyomavirus (JCPyV) in an animal model [18,19]. Alternative splicing is a hallmark of cancer that has recently been shown to be an important alternative oncogene and oncogene signaling pathway activation mechanism in tumors exhibiting low mutation rates [20,21,22], which would include PitNETs. Within this context, normal pituitary cells and PitNETs express the neurotrophin receptor tropomyosin receptor kinase A (TrkA) [9,23,24], which exhibits oncogenic alternative *TrkAIII* splicing in human neuroendocrine neuroblastomas (NBs), Merkel Cell polyomavirus (MCPyV) positive Merkel cell carcinomas, cutaneous malignant melanomas, and acute myeloid leukemia [25,26,27,28,29].

The oncogenic alternative *TrkAIII* splice variant (GeneBank OP866787.1) is characterized by *NTRK1/TrkA* exons 6, 7, and 9 skipping, and it was first identified in human NBs in association with post-therapeutic relapse and advanced-stage metastatic disease [25,28]. The variant TrkAIII receptor lacks the extracellular D4 IG-C1 domain and several N-glycosylation sites that are required for fully spliced TrkA receptor cell surface expression and prevention of ligand-independent activation [30,31,32]. These omissions result in the intracellular re-localization of TrkAIII to pre-Golgi membranes, centrosomes, and mitochondria, where TrkAIII exhibits ligand-independent, cell cycle-regulated, stress-regulated, and doxorubicin-induced intracellular activation [25,33,34,35,36]. The intracellular activation of TrkAIII results in pro-survival phosphoinositide 3-kinase (PI3K)/Akt signaling, increased expression of B-cell lymphoma 2 (Bcl-2), myeloid cell leukemia sequence 1 (Mcl-1) and superoxide dismutase 2 (SOD2), a pro-angiogenic expression equilibrium between matrix metalloproteinase-9 (MMP-9)/vascular endothelial cell growth factor (VEGF)/thrombospondin 1 (Tsp1), centrosome amplification, stress-regulated metabolic adaptation, a modified unfolded protein response (UPR), and a more anaplastic stem cell-like phenotype [25,30]. TrkAIII oncogenic activity (NIH3T3 cell transformation and promotion of primary and metastatic tumorigenicity in NB models), furthermore, is similar to that of the TrkA-fusion oncogene *TrkT3* [25], confirming TrkAIII to be a splice variant oncogenic equivalent of *TrkA-fusion* oncogenes and also the engineered D4 domain-deleted *TrkA* oncogene [31,37,38].

Alternative *TrkAIII* splicing in NB cells is promoted by hypoxia, agents that cause endoplasmic reticulum (ER), Ca^2+^, redox, and nutrient stress, and by the simian vacuolating polyomavirus virus 40 (SV40) large T-antigen [25,30,36]. Considering that PitNETs exhibit low mutation rates, express TrkA, and associate with conditions linked to alternative splicing and polyomavirus infection, we investigated alternative *TrkAIII* splicing as a potentially targetable participant in PitNET pathogenesis and progression. Overall, the data support a role for alternative *TrkAIII* splicing in PitNET pathogenesis and progression, potentially involving hypoxia, and identify TrkAIII as a novel potential therapeutic target in refractory PitNETs.

## 2. Materials and Methods

### 2.1. Patients and Tumors

PitNETs from 53 patients were surgically removed at the Neuromed Institute (Pozzili, Italy). Prior to surgery, all patients were characterized for bio-clinical evidence of hormone hypersecretion and, by Magnetic Resonance Imaging (MRI), for macroscopic tumor characteristics. In total, 24 patients were clinically diagnosed with functioning PitNETs (6 prolactinomas, 13 acromegaly, 2 central hyperthyroidism, and 4 Cushing’s disease), and the remaining 29 were diagnosed with clinically non-functioning tumors. With the exception of a young female with a micro-prolactinoma, all other patients had macro-tumors (maximal diameter > 10 mm). Tumor invasion of surrounding structures (cavernous sinus/sphenoid sinus/bone/dura) was identified by pre-operative MRI and surgical findings. Overall, 26 of 53 PitNETs and 4 of 6 recurrent PitNETs were invasive (49%), of which 2 were aggressive and 1 was metastatic. Routine immunohistochemical (IHC) pathological tumor classification and diagnosis were performed in accordance with European Pituitary Pathology Group proposals [39], using primary antibodies directed against pituitary hormone, transcription factors, and Ki67 (MIB1clone). Analyses were performed using an Ultraview DAB detection kit (Roche Diagnostics Int.; Rotkeuz, Switzerland) in an automatic VENTANA Benchmark ultra XT IHC/ISH System, as directed (Roche Diagnostics Int.; Rotkeuz, Switzerland).

The PitNETs examined in this study were classified according to their lineage of origin as follows: 24 PIT1, 24 SF1, and 5 TPIT-positive tumors, the details for which are provided in Table 1. For molecular studies, surgical tumor fragments were immediately placed in RNA*later*^TM^ nucleic acid stabilizing solution, as directed (Ambion^®^, Life Technologies, Monza, Italy), and frozen at −80 °C prior to nucleic acid purification. In some cases, slide-mounted 4 μm FFPE PitNET tissue sections were also provided for confocal immunofluorescence analysis. This study was approved by the Neuromed Institute Internal Review Board, as a part of the Biopit study (Biopit 270423), and performed according to Helsinki declarations. Written informed consent was obtained from patients, with the exception of a minority of archived RNAs from patients lost to follow-up.

### 2.2. Antibodies and Reagents

Mouse monoclonal anti-human TrkA carboxyl-terminus (cod. SC-7268 (B3), 200 µg/mL) antibody was from Santa Cruz Biotechnology (Dallas, TX, USA) and recognizes both *fs*-TrkA and TrkAIII [25,26,27]. Rabbit monoclonal anti-human Y490-phosphorylated TrkA antibody (cod. 9141; 36 µg/mL) was from Cell Signaling Technology (Danvers, MA, USA) and recognizes both phosphorylated *fs*-TrkA and TrkAIII [25,26,27]. Secondary Alexa Flour 488-labeled donkey anti-rabbit and Alexa Fluor donkey anti-mouse antibodies were from Life Technologies (1 mg/mL) (Fortis, Waltham, MA, USA). Prolong^TM^ Gold anti-fade reagent with DAPI was from Invitrogen (Thermo-Fisher Scientific, Waltham, MA, USA).

### 2.3. RNA Extraction and Reverse Transcriptase Polymerase Chain Reaction

Total RNAs were extracted from tissues using Trizol, according to the manufacturer’s instructions (Life Technologies, Monza, Italy). Briefly, tumor tissues were homogenized in 1 mL of Trizol, and resulting supernatants were mixed with chloroform and centrifuged to obtain phase separation. The upper phase was recovered and washed in isopropanol, RNAs were then precipitated in 75% ethanol and centrifuged at 14,000× *g* in an Eppendorf microfuge at 4 °C, and RNA pellets were resuspended in 20μL of RNase/DNAse-free water. RNA purity and concentrations were evaluated in a nanodrop spectrophotometer, as directed (Thermo Fisher Scientific, Carlsbad, CA, USA). Purified RNAs were reverse-transcribed using a Wonder RT transcription kit, as directed (Euroclone, Pero, Italy), and reverse transcription reactions, at various dilutions, were subjected to RT-PCR, using the primers and conditions detailed in Table 2. All RT-PCRs were performed in duplicate and repeated at least 2 times. For densitometric analysis, 1.5% agarose gels were digitally photographed and images analyzed by Image J software (ImageJ bundled with Java 1.8.0_172), with inter-gel comparisons performed using common 18S rRNA RT-PCR product and DNA ladder standards, where appropriate.

### 2.4. Tumor DNA Purification

Tumor DNA (tDNA) was extracted from 8 PRL PitNETs using Quick-DNA Miniprep Plus Kit, as directed (Zymo Research, Irvine, CA, USA). DNA quality was checked by 0.8% agarose gel electrophoresis and PCR amplification for the housekeeping gene GAPDH [40].

### 2.5. DNA Sequencing

For DNA sequencing, *TrkA* exon 1-8, *TrkA* exon 8-17, and SF3B1 RT-PCR products (cDNA and tDNA) were purified from ethidium bromide-stained agarose gels, using a Jet Quick gel extraction spin kit, as directed (Genomed, Harrow, UK), cleaned using a EuroSAP PCR enzymatic Clean-Up kit, as directed (Euroclone, Milan, Italy), and PCR amplified using the primers detailed in Table 2 and the BigDye Terminator V.2.1. Cycle Sequencing kit, as directed (Thermo-Fisher Scientific, Carlsbad, CA, USA). Re-amplified products were sequenced by double-stranded Sanger sequencing, in a mono-capillary DNA sequencer (Genetic Analyzer 3500, Thermo-Fischer Scientific, CA, USA).

### 2.6. Indirect IF

FFPE sections (4 µm) were de-paraffinized, re-hydrated, and processed for antigen retrieval by incubation in 0.01 M sodium citrate buffer (pH 6.0) for 20 min at 98 °C. Sections were blocked in blocking solution (1 × PBS, 5% BSA, 0.1% Triton X-100), incubated overnight at 4 °C with mouse monoclonal anti-human TrkA (B3, 1:100 dilution in 1 × PBS, 1% BSA, 0.1% Tx100) and rabbit monoclonal anti-human Y490-phosphorylated TrkA (pY490-TrkA, 1:100 dilution, in 1 × PBS, 1% BSA, 0.1% Triton X-100) primary antibodies, washed extensively in PBS, and then incubated with appropriate fluorochrome-conjugated Alexa Fluor secondary antibodies (diluted 1:1000 in 1 × PBS) for 2 h at 37 °C. Slides were then washed and counterstained with Bisbenzimide nuclear dye (Hoechst, Thermo Fisher Scientific, CA, USA), and images were acquired under scanning confocal microscopy (Leica TCS SP5 II).

### 2.7. Statistical Analysis

Data are expressed as median (range) and were statistically analyzed using the following non-parametric tests: Mann–Whitney U and Kruskal–Wallis tests for comparisons of continuous variables between 2 or 3 groups, respectively, and Spearman’s correlation test. *p* values < 0.05 were considered significant.

## 3. Results

Table 1 lists the specifics of each patient and tumor. The corresponding case numbers (n.) are utilized throughout the manuscript. In this cohort, 21 of the 24 PIT1 PitNETs were functioning tumors and included 6 functioning and 1 silent lactotrophs (PRLs), 9 functioning and 1 silent somatotrophs (GHs), 4 functioning mixed PRL/GHs, 2 functioning thyrotrophs (TSHs), and 1 hormone-negative tumor. Eleven (45.8%) PIT1 PitNETs were invasive. All 24 SF1/gonadotroph PitNETs were clinically non-functioning and included 18 hormone-positive (FSH and/or LH) and 6 pure SF1 tumors. Fourteen (58.3%) SF1 PitNETs were invasive. The majority of TPIT PitNETs (4/5) were functioning, 1 was a silent ACTH-secreting tumor, and 3/5 were invasive. *TrkAIII* mRNA was detected in almost all PitNETs, with the exception of three non-invasive PIT1 and one non-invasive SF1 PitNETs (see Table 1).

### 3.1. TrkAIII Was the Only in-Frame Alternative TrkA Splice Variant Expressed in PitNETs

**Figure 1:**
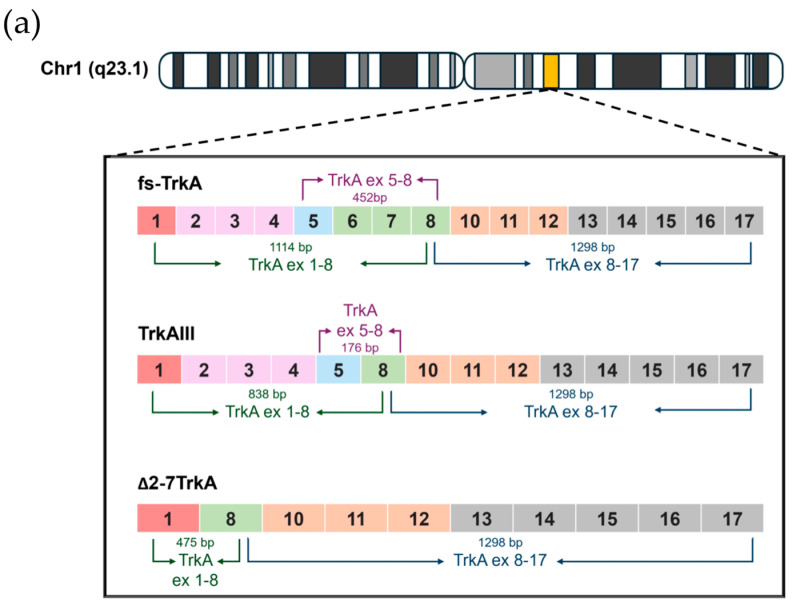

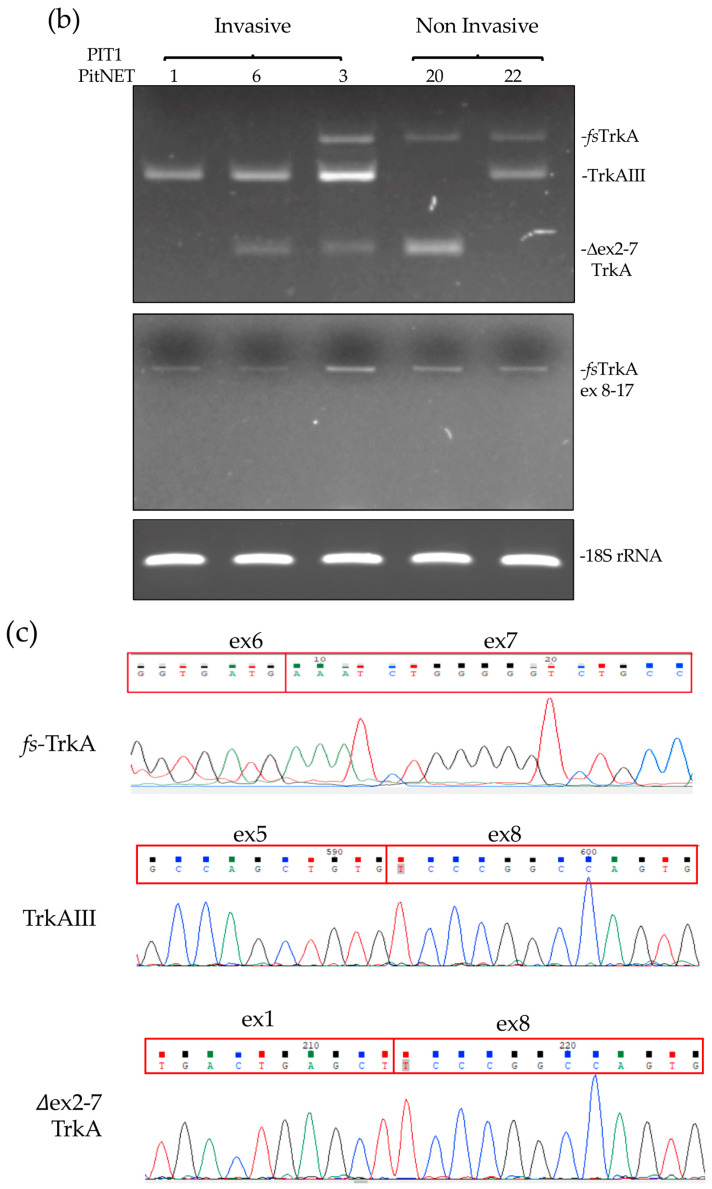
(**a**) Schematic diagram of chromosomal *NTRK1/TrkA* gene localization, *fs-TrkA*, *TrkAIII*, and Δ*2-7TrkA* exon structures and exon 1–8 and 8–17 RT-PCR amplicons, in base pairs (bps). (**b**) Representative RT-PCRs demonstrating *fs-TrkA*, *TrkAIII*, and Δ*2-7 TrkA* products generated using primers spanning *TrkA* exons 1 through 8 and single products generated using primers spanning TrkA exons 8 through 17, sequence characterized as containing all *NTRK1/TrkA* exons 8 through 17, in cDNAs from invasive (n.1, 3, and 6) and non-invasive (n.20 and 22) PIT1 PitNETs. (**c**) Representative DNA sequences demonstrating the *fs-TrkA* exon 6–7, *TrkAIII* exon 5–8, and Δ*ex2-7 TrkA* exon 1–8 splice junctions in purified RT-PCR products in invasive PIT1 PitNET (n.3).

RT-PCR, using primers spanning *NTRK1/TrkA* exons 1 through 8, detected three products in PitNET cDNAs that were sequence characterized as the fully spliced *TrkA* transcript *fs-*TrkA; the exons 6 and 7 skipped transcript *TrkAIII*, and the exons 2–7 skipped transcript Δ*2-7TrkA* (Figure 1a–c). PitNETs were also analyzed using primers spanning *NTRK1/TrkA* exons 8 through 17, which produced single products (Figure 1b, middle panel) that were sequence characterized as containing fully spliced *TrkA* exons 8 through 17 (not shown). *Fs-TrkA* and *TrkAIII* were the only in-frame splice variant mRNAs expressed in PitNETs. The Δ*2-7TrkA* splice variant was sequence characterized as a nonsense mRNA (Figure 1c). This variant exhibits a frameshift at the novel exon 1/8 splice junction that results in a premature TGA stop codon at position 1039–1041 (*fs-TrkA* numeration) (this study) and in [26,27].

Due to limited PitNET RNA availability, based on recommended PCR amplicon sizes and to improve the semi-quantitative evaluation of *TrkAIII* to *fs-TrkA* RT-PCR ratios, specific primers spanning *NTRK1/TrkA* exons 5 through 8 were employed. These primers produce 452 bp *fs*-*TrkA* and 176 bp *TrkAIII* amplicons within single RT-PCR reactions. This primer set did not introduce an amplification bias in either *fs-TrkA* or *TrkAIII* amplicons in regular PCR reactions containing 1 to 1, 1 to 4, and 4 to 1 femtomolar mixtures of recombinant *fs-TrkA* and *TrkAIII* cDNAs.

RT-PCR using this primer set detected *TrkAIII* mRNA expression in all invasive PitNETs regardless of lineage, and also detected *TrkAIII* mRNA in ≈86% of non-invasive PitNETs, comprising 10 PIT1, 12 SF1, and 2 TPIT tumors. This primer set also confirmed exclusive *TrkAIII* mRNA expression in two invasive PIT1 and one invasive SF1 PitNETs, as well as in three invasive and one non-invasive TPIT PitNETs. In contrast, exclusive *fs-TrkA* mRNA expression was only detected in three non-invasive PIT1 and one non-invasive SF1 PitNETs but not in any invasive PitNET.

In semi-quantitative densitometric RT-PCR analyses, *TrkAIII* to *fs-TrkA* RT-PCR ratios in individual invasive PitNETs ranged from 21.7% to 100%, and in non-invasive PitNETs they ranged from 0% to 91.8%. *TrkAIII* to *fs-TrkA* ratios were significantly higher in invasive compared to non-invasive PitNETs (median 61.1% versus 43%: *p* = 0.048). When grouped into lineages, *TrkAIII* to *fs-TrkA* ratios were significantly higher in invasive PIT1 (range 34.4% to 100%, median 60.2%) compared to non-invasive PIT1 (range 0% to 59%, median 32.3%, *p* = 0.035) PitNETs. In contrast, ratios were similar in both invasive and non-invasive SF1 PitNETs (median 52.1% vs. 49.2%, *p* = 0.238). Invasive TPIT PitNETs all exhibited exclusive (100%) *TrkAIII* mRNA expression but were too few for statistical comparison to the two non-invasive TPIT PitNETs. Kruskal–Wallis statistical analysis confirmed that *TrkAIII* to *fs-TrkA* ratios were significantly different between the three PIT1, SF1, and TPIT lineages (*p* = 0.007).

**Figure 2:**
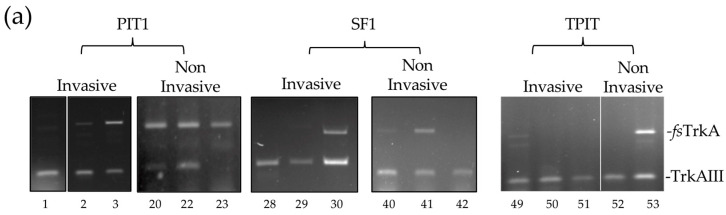

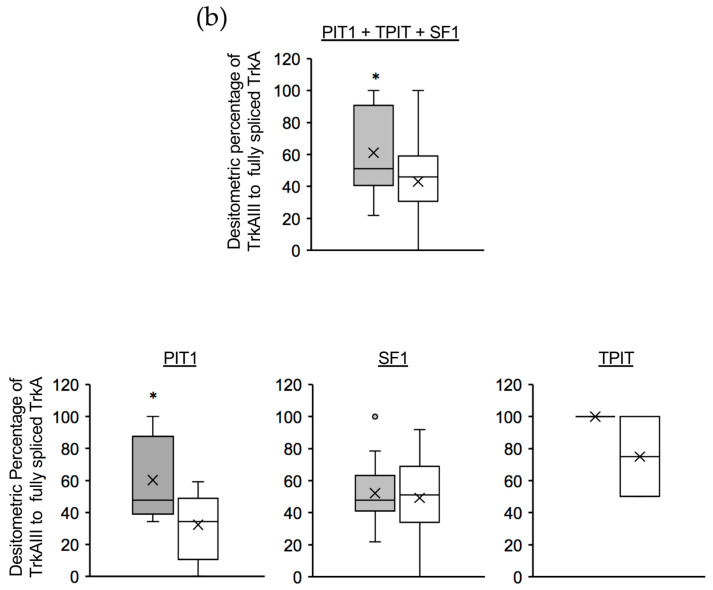
(**a**) RT-PCRs demonstrating relative levels of *TrkAIII* to *fs-TrkA* expression in cDNAs from representative invasive and non-invasive PIT1, SF1, and TPIT PitNETs. (**b**) Box plots demonstrating densitometric comparisons of percentage *TrkAIII* to *fs-TrkA* ratios in all invasive (grey) and non-invasive (white) PitNETs (PIT1 + TPIT + SF1), as well as PitNETs grouped into PIT1, SF1, and TPIT lineages. Exclusive TrkAIII expression (100%) was detected in all 3 invasive TPIT PitNETs (* = *p* < 0.05, x represents mean values, circles refer to outliers).

With respect to aggressive and metastatic PitNET behavior, it is worth noting that aggressive invasive PIT1 PitNET (n.1) exhibited exclusive TrkAIII expression (Figure 2a), whereas the invasive metastatic PIT1 PitNET (n.11), reported previously to be responsive to immunotherapy [41], exhibited a ≈65 to 35% *fs-TrkA to TrkAIII* RT-PCR ratio.

### 3.2. TrkAIII mRNA Expression Associates with IF Immunoreactivity Consistent with Intracellular TrkAIII Activation

**Figure 3:**
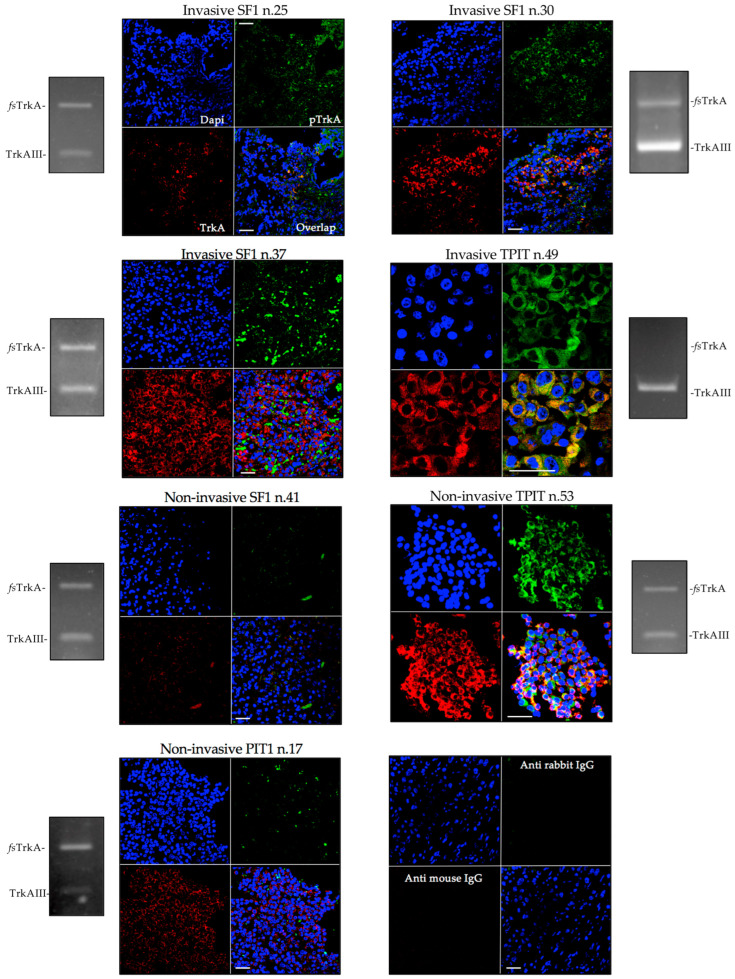
Confocal IF micrographs, demonstrating overlapping (orange/yellow) TrkA (red) and Y490 phosphorylated TrkA (green) isoform(s) immunoreactivity in invasive SF1 PitNETs (n.25, 30, and 37), invasive TPIT PitNET (n.49), and non-invasive SF1 PitNET (n.41), TPIT PitNET (n.53), and PIT1 PitNET (n.17). Bottom right panels demonstrate background secondary antibody immunoreactivity, and nuclei are colored blue (bar = 100 μm).

Due to limited tissue availability, confocal immunofluorescence for overlapping non-phosphorylated and phosphorylated TrkA isoform(s) immunoreactivity was assessed in a representative subgroup of four invasive (n.25, 30, 37, 49) and three non-invasive (n.17, 41, 53) PitNETs and compared to individual *fs-TrkA* and *TrkAIII* RT-PCR ratios (Figure 3). Using antibodies that recognize both *fs*-TrkA and TrkAIII [21,22,23], the highest levels of overlapping TrkA and phosphorylated TrkA isoform(s) immunoreactivity were detected in an invasive TPIT PitNET (n.49) exhibiting exclusive TrkAIII expression and in a non-invasive TPIT PitNET (n.53) exhibiting an approximately equal *fs-TrkA* and *TrkAIII* RT-PCR expression. Lower levels of overlapping immunoreactivity were also observed in three invasive SF1 PitNETs (n.25, 30, 37) exhibiting different *TrkAIII to fs-TrkA* RT-PCR ratios. In contrast, immunoreactivity was close to the background in a non-invasive SF1 PitNET (n.41) exhibiting an approximately equal *fs-TrkA to TrkAIII* RT-PCR ratio and was restricted to the non-phosphorylated TrkA isoform(s) in a non-invasive PIT1 PitNET (n.17) exhibiting a predominant *fs-TrkA* to *TrkAIII* RT-PCR ratio.

These findings demonstrate that PitNETs exhibiting exclusive *TrkAIII* mRNA expression, predominant *TrkAIII*, or approximately equal *TrkAIII* to *fs-TrkA* RT-PCR ratios show evidence of intracellular TrkA isoform(s) expression and phosphorylation. The strongest evidence for intracellular TrkAIII expression and activation comes from the overlapping immunoreactivity observed in two invasive PitNETs (n.30 and 49) exhibiting exclusive or near-exclusive *TrkAIII* mRNA expression.

### 3.3. Enhanced Alternative TrkAIII Splicing in Invasive PIT1 PitNETs Associates with Increased HIF2α mRNA Expression

**Figure 4:**
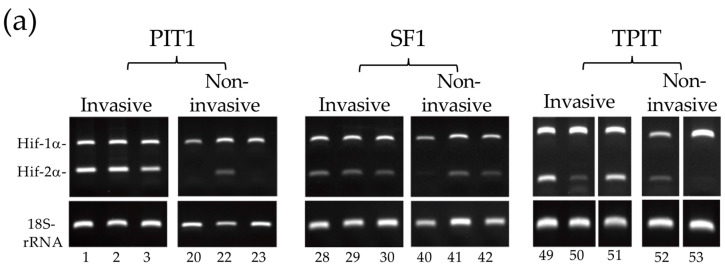

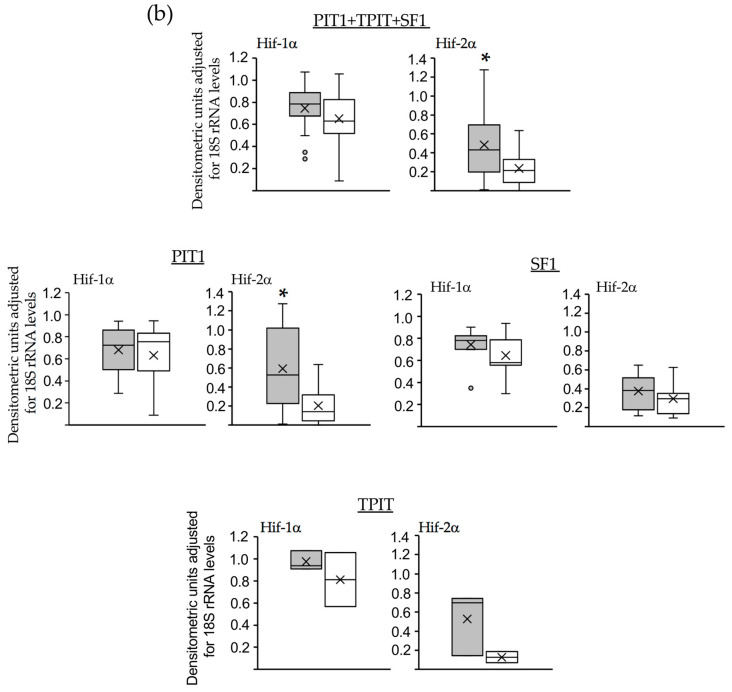
(**a**) RT-PCR demonstrating relative *HIF-1α* (150 bp) and *Hif-2α* (121 bp) RT-PCR products in representative invasive and non-invasive PIT1, SF1, and TPIT PitNETs, run on the same gel for comparison. (**b**) Box plots demonstrating comparative HIF-1α and Hif-2α RT-PCR levels in all invasive (grey) and non-invasive (white) PitNETs (PIT1 + TPIT + SF1) and in PitNETs grouped into PIT1, SF1, and TPIT lineages (* *p* < 0.05, x represents mean values, circles refer to outliers).

Potential hypoxia involvement in PitNET alternative *TrkAIII* mRNA splicing was assessed by RT-PCR analysis of *HIF2α* and *HIF1α* expression in cDNAs from 50 *TrkAIII* mRNA expressing PitNETs, for which RNAs were available. Densitometric RT-PCR analysis revealed that invasive PitNETs expressed significantly higher *HIF2α* levels than non-invasive PitNETs (*p* = 0.0028) (Figure 4). *HIF-2α* levels were also significantly higher in invasive compared to non-invasive PIT1 PitNETs (*p* = 0.0198) but did not distinguish between invasive and non-invasive SF1 PitNETs (*p* = 0.238). Invasive TPIT PitNETs, although too few for lineage-restricted statistical comparisons, also exhibited high levels of *HIF2a* expression compared to non-invasive counterparts. Kruskal–Wallis analysis did not detect a significant difference between *HIF2α* expression in combined invasive and non-invasive PIT1, SF1, and TPIT lineages (*p* = 0.849). Although alternative TrkAIII splicing and HIF2α were significantly elevated in invasive PIT1 PitNETs, Spearman’s correlation coefficient analysis failed to confirm a direct correlation between *HIF2α* levels and *TrkAIII* to *fs-TrkA* RT-PCR ratios in individual invasive (*p* = 0.8) or non-invasive (*p* = 0.076) PIT1 PitNETs.

*HIF1α* levels were not significantly different in invasive compared to non-invasive PitNETs (*p* = 0.168), nor in invasive and non-invasive PIT1 (*p* = 0.78) or SF1 (*p* = 0.15) PitNETs, and were also similar in invasive and non-invasive TPIT PitNETs (Figure 4a,b).

These data confirm an association between elevated *HIF2α* expression and invasive PitNETs, especially invasive PIT1 PitNETs, and they also confirm an association but not a direct correlation between elevated *HIF2α* levels and elevated alternative *TrkAIII* splicing in PIT1 PitNETs.

### 3.4. Alternative TrkAIII Splicing in PitNETs Does Not Associate with Hotspot SF3B1 Mutations or De-Regulated SF3B1, SRSF2, U2AF1 Expression

**Figure 5:**
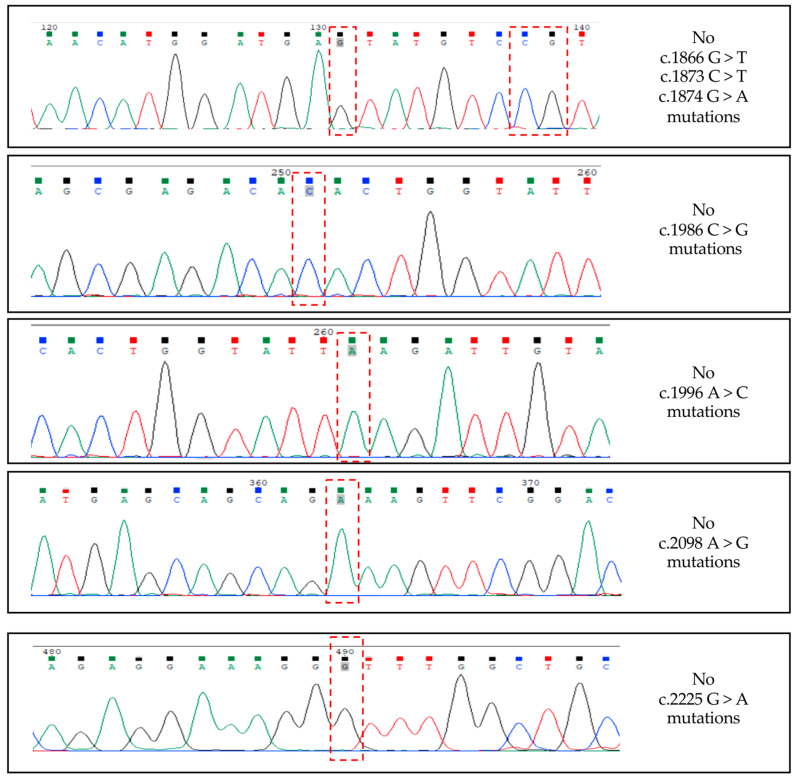
Representative **(a)** SF3B1cDNA sequences in PIT1 PitNET 1, demonstrating the absence of PitNET-associated hotspot SF3B1 c.1886, c.1873. c.1874, c.1986, c.1996, c.2098, and c.2225 mutations (red hatched boxes identify hotspot mutation sites).

Considering that lactotroph PitNETs associate with somatic hotspot *SF3B1* mutations [14,15,16], tumor DNAs (tDNA) from 6 and cDNAs from 22 *TrkAIII* RT-PCR positive lactotroph PitNETs were evaluated for the presence of hotspot SF3B1 c.1866 G > T; c. 1873 C > T; c. 1874 G > A; c.1986 C > G; c.1996 A > C; c.2098 A > G (cDNA/tDNA), and c.2225 G > A (cDNA only) mutations. None of these mutations were detected in any of the PitNET tDNAs or cDNAs examined (Figure 5, representative example).

**Figure 6:**
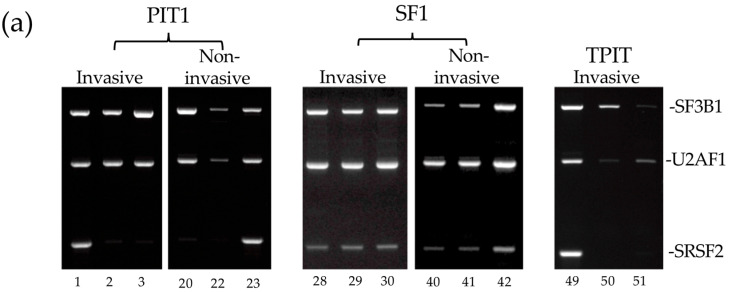

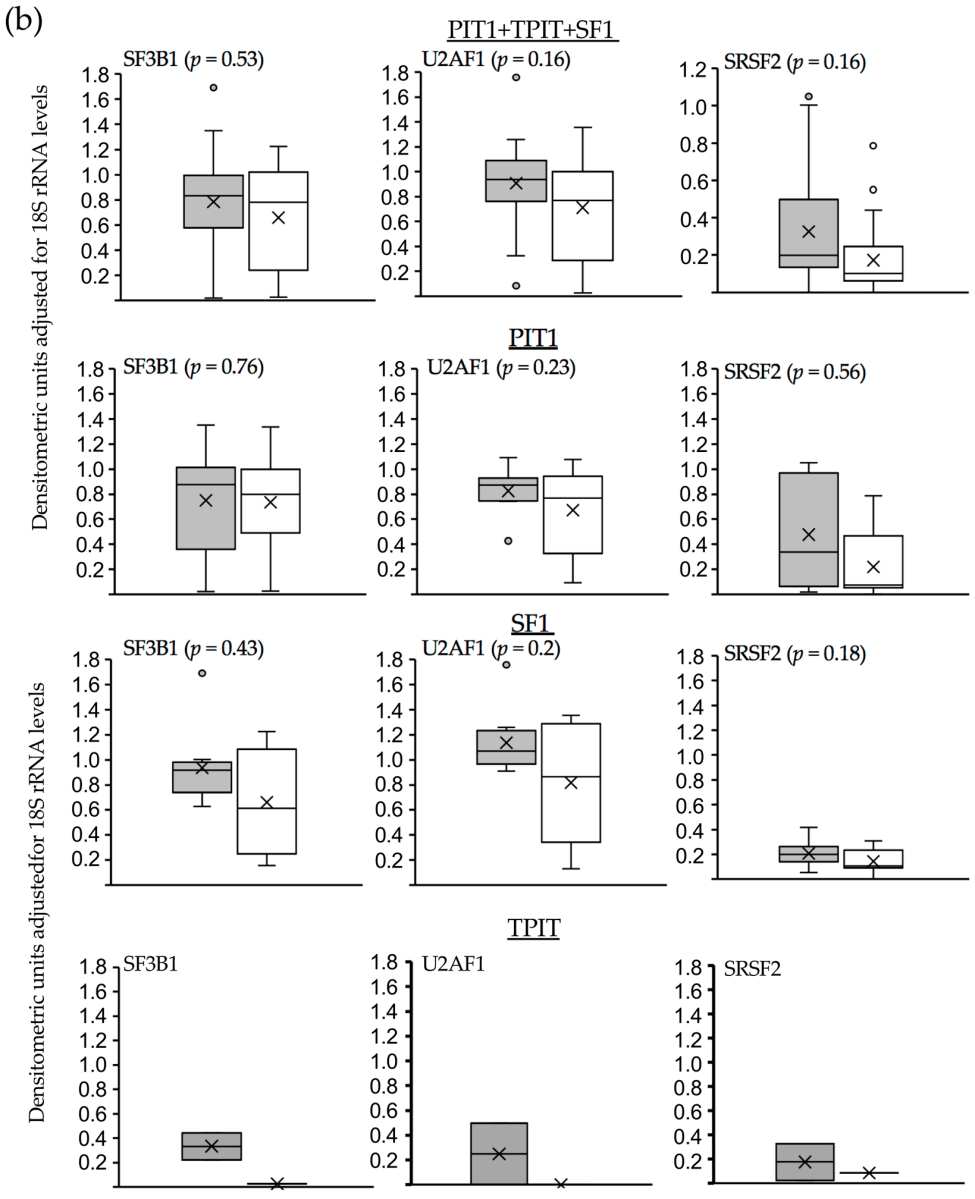
(**a**) RT-PCRs demonstrating SF3B1 (693 bp), U2AF1 (606 bp), and SRSF2 (408 bp) products in representative invasive and non-invasive PIT1, SF1, and TPIT PitNET cDNAs, run on the same gel for comparison. (**b**) Box plots demonstrating densitometric comparisons of SF3B1, U2AF1, and SRSF2 levels in all invasive (grey) and all non-invasive (white) PitNETs (PIT1 + TPIT + SF1) and in PitNETs grouped into PIT1, SF1, and TPIT lineages (Mann–Whitney *p* values are provided in brackets, x represents mean values, circles refer to outliers).

In light of a report linking dysregulated splice factor expression to PitNET pathogenesis and aggressive behavior [17], PitNETs were also examined for alterations in *SF3B1, U2AF1*, and *SRSF2* splice factor mRNA expression, by densitometric RT-PCR. No significant variations in *SF3B1*, *U2AF1*, or *SRSF2* expression levels were detected between invasive and non-invasive in PitNETs, or individual PIT1 and SF1 lineages. This implies that variations in alternative *TrkAIII* splicing in PitNETs are unlikely to depend upon altered *SF3B1*, *U2AF1*, or *SRSF2* mRNA expression (Figure 6).

### 3.5. PitNET Alternative TrkAIII Splicing Does Not Associate with Unconventional Xbp1 Splicing or JCPyV Large T Antigen mRNA Expression

**Figure 7:**
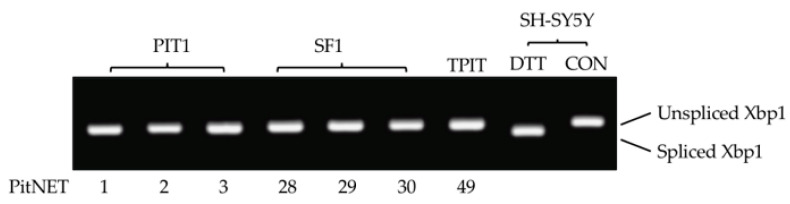
RT-PCR detection of unconventional Xbp1 splicing (spliced Xbp1) in DTT-treated but not in untreated (CON) SH-SY5Y cells or representative examples of PIT1 (n.1, 2, and 3), SF1 (n.28, 29, and 30) and TPIT (n.49) PitNET cDNAs.

Agents that activate the UPR also promote alternative *TrkAIII* splicing in NB cells [25,30,33,36]. A potential role for UPR activation in PitNET alternative TrkAIII splicing was examined by RT-PCR analysis of unconventional Xbp1 splicing, which serves as an index of UPR activation [42]. Unconventional Xbp1 splicing, detected in DTT-treated (positive control) but not untreated (negative control) SH-SY5Y cDNAs, was not detected in any of the 50 PitNET cDNAs analyzed (Figure 7).

JCPyV polyomavirus infection has been implicated in PitNET pathogenesis [18,19]. JCPyV *large T-antigen* mRNA expression, as a potential indicator of JCPyV infection, was assessed in PitNETs, by RT-PCR. JCPyV *large T-antigen* mRNA expression was not detected in 45 PitNETs exhibiting alternative *TrkAIII* splicing, suggesting that JCPyV infection is unlikely to be responsible for alternative *TrkAIII* splicing in this PitNET cohort.

## 4. Discussion

In this study, we report that alternative *TrkA* mRNA splicing, limited to *NTRK1*/*TrkA* exons 1 through 8, is highly prevalent in PitNETs, regardless of their lineage of origin. We validate that PitNETs express three alternative splice variants *fs-TrkA*, *TrkAIII*, and Δ*2-8TrkA* and that *TrkAIII* is the only in-frame, tyrosine kinase-domain encoding, potentially oncogenic alternative to *fs*-TrkA. Although alternative *TrkAIII* mRNA splicing was detected in both invasive and non-invasive PitNETs, it was significantly elevated in invasive tumors, particularly in the invasive PIT1 PitNET group.

These data extend earlier findings of *NTRK1/TrkA* exons 1–8-restricted alternative TrkA splicing in NBs, MCPyV positive Merkel cell carcinomas, and cutaneous malignant melanomas [25,26,27,28] and indicate that *NTRK1/TrkA* exons 2 through 7 are more prone to alternative splicing. Furthermore, PitNETs expressed only *TrkAIII* and Δ*2-7TrkA* variants, in contrast to Merkel cell carcinomas and melanomas, which express several alternative exons 2–7 TrkA splice variants [26,27]. Additionally, a unique feature of PitNEts amongst these tumor types was the frequent detection of predominant and occasionally exclusive *TrkAIII* mRNA expression.

The detection of *TrkAIII* mRNA expression in invasive and non-invasive PitNETs and immunoreactivity is consistent with intracellular TrkAIII activation in PitNETs exhibiting exclusive or near-exclusive *TrkAIII* mRNA expression, suggesting that TrkAIII participates in different stages of PitNET pathogenesis and progression. Interestingly, exclusive and predominant *TrkAIII* mRNA expression was more common in invasive PitNETs, whereas exclusive *fs-*TrkA expression was only detected in non-invasive PitNETs. In accordance with this, alternative *TrkAIII* splicing was significantly higher in invasive compared to non-invasive PitNETs. However, when grouped according to lineage, it was only significantly higher in invasive compared to non-invasive PIT1 PitNETs. In contrast, invasive and non-invasive SF1 PitNETs exhibited similar levels of alternative *TrkAIII* splicing. Although TPIT PitNETs were too few for statistical comparisons within the group, it is remarkable that all three invasive cases exhibited exclusive *TrkAIII* mRNA expression. Overall, these findings indicate that divergent factors may influence alternative *TrkAIII* splicing in different PitNET lineages. Furthermore, they suggest enhanced potential for TrkAIII involvement in invasive PIT1 PitNET behavior and similar potential for involvement in both invasive and non-invasive SF1 PitNET behavior. Alternative *TrkAIII* splicing should, therefore, be added to the growing network of molecular changes associated with PitNET pathogenesis and progression [5,10,43].

The strongest evidence for TrkAIII involvement in PitNET pathogenesis and progression can be detected in high-level over-lapping non-phosphorylated and phosphorylated TrkA isoform(s) immunoreactivity in PitNETs exhibiting exclusive or near-exclusive TrkAIII mRNA expression. Lower levels of overlapping immunoreactivity were also detected in three invasive SF1 PitNETs exhibiting variable levels of alternative *TrkAIII* splicing. In contrast, immunoreactivity was barely detectable in two non-invasive SF1 (n.41) and PIT1(n.17) PitNETs exhibiting predominant *fs-TrkA* to *TrkAIII* RT-PCR ratios. Overall, these findings support a functional relationship between *TrkAIII* mRNA expression and intracellular TrkA isoform(s) expression and activation, including the TrkAIII oncoprotein. This is in line with reports that PitNETs and pituitary cell types exhibit heterogeneous TrkA expression, which has previously limited research interest in the potential significance of TrkA in these tumors [24,44].

Hypoxia promotes alternative *TrkAIII* splicing in neural crest progenitors, neural stem cells, and NB cells [25,36]. PitNETs also show activated hypoxia responses, including HIF1α-RSUME-VEGF pathway activation, which is involved in PitNET progression and represents a current therapeutic target in refractory disease [6,7,10,43,44,45]. Because PitNET protein extracts were not available, the investigation into potential hypoxia participation in PitNET alternative *TrkAIII* splicing was restricted to RT-PCR comparisons with *HIF1α* and *HIF2α* expression. Elevated alternative *TrkAIII* splicing was linked to significantly higher levels of *HIF2α* but not *HIF1α* mRNA expression in invasive PIT1 PitNETs, suggesting a potential role for hypoxia in PitNET alternative TrkAIII splicing. This finding also identifies HIF2α as a novel potential marker of invasive PIT1 PitNET behavior. However, no significant correlation could be found between *HIF2α* expression and alternative *TrkAIII* mRNA splicing in individual PIT1 PitNETs. This does not rule out a role for hypoxia in alternative *TrkAIII* splicing, since all PitNETs expressed *HIF1α* mRNA, and HIF1α is involved in the PitNET hypoxia response [6,45,46,47]. Hypoxia also stimulates HIF1α and HIF2α protein expression at the post-transcriptional level [48]. Notably, NB cells are one of the few cell types that show *HIF2α* transcriptional sensitivity to hypoxia [49] and also exhibit hypoxia-regulated alternative *TrkAIII* splicing [25,36], revealing a similarity between NBs and PIT1 PitNETs, potentially based on a common neural crest cell origin [50,51,52,53,54,55].

In relation to potential molecular mechanisms that could promote *TrkAIII* splicing in PitNETs, hotspot mutations in splicing factor *SF3B1* have been reported in lactotroph PitNETs and have been shown to induce aberrant splicing [14,15,16,17]. However, hotspot *SF3B1* c.1866 G > T, c.1873 C > T, c.1874 G > A, c.1986 C > G, c.1996 A > C, c.2098 A > G and c.2225 G > A mutations were not detected in any of the *TrkAIII* mRNAs expressing PitNETs analyzed, excluding potential involvement.

PitNET pathogenesis and aggressive behavior have also been linked to dysregulated splice factor expression [17]. PitNET RNA availability limited the examination of dysregulated splicing factors in this study to *SF3B1*, *SRSF2*, or *U2AF1*. These were selected for analysis based on observations that SF3B1 regulates splicing in PitNET cells, and both SRSF2 and U2AF1 are differentially expressed in different PitNET lineages [17]. No significant variations in *SF3B1*, *SRSF2*, and *U2AF1* expression were detected between invasive and non-invasive PitNETs, either as a whole or grouped according to PIT1 and SF1 lineages. Furthermore, altered *SF3B1*, *SRSF2*, and *U2AF1* expression did not correlate with enhanced alternative *TrkAIII* splicing in invasive PIT1 PitNETs. However, since the splicing machinery is complicated, we do not rule out the potential involvement of other splicing factors dysregulated in PitNETs [17].

Agents that cause ER stress and activate the UPR also promote alternative TrkAIII mRNA splicing in NB cells [25,30,33,36]. In this investigation, unconventional Xbp-1 splicing, which serves as an index of UPR activation [42], was assessed in order to evaluate the relationship between the UPR and PitNET alternative TrkAIII splicing. No PitNETs displaying *TrkAIII* expression exhibited unconventional Xbp-1 splicing, potentially ruling out a role for the UPR. This was surprising, considering that hypoxia triggers UPR activation [56] and PitNETs exhibit activated hypoxia responses [6,45,46,47]. It is unclear if this may reflect a malfunctioning IRE1/Xbp1 arm of the UPR, TrkAIII modification of the UPR [30], or some other mechanism.

Finally, an examination of JCPyV large T antigen expression in PitNETs was prompted by a possible role for JCPyV infection in PitNET pathogenesis [18,19], by SV40 large T antigen promotion of alternative *TrkAIII* splicing in NB cells, and by alternative *TrkAIII* splicing association with MCPyV large T antigen expression in Merkel cell carcinomas. JCPyV *large T antigen* expression, however, was not detected in any of the PitNETs examined, suggesting that JCPyV is not involved in PitNET alternative TrkAIII splicing.

## 5. Conclusions

In conclusion, this study reveals that alternative *TrkAIII* mRNA splicing is common in all PitNET lineages and is significantly more pronounced in invasive PitNETs, especially invasive PIT1 PitNETs. It also reveals that significant increases in alternative *TrkAIII* mRNA splicing are associated with significantly elevated *HIF2α* mRNA expression in invasive PIT1 PitNETs, linking alternative TrkAIII splicing to the hypoxia response. We also verify that exclusive TrkAIII mRNA expression is associated with immunoreactivity consistent with intracellular expression and activation of the TrkAIII oncoprotein and that TPIT PitNETs appear to be especially susceptible to exclusive *TrkAIII* mRNA expression and intracellular TrkAIII activation. We conclude, therefore, that alternative *TrkAIII* mRNA splicing, leading to intracellular expression and activation of the TrkAIII oncoprotein, is likely to participate in PitNET pathogenesis and progression. TrkAIII may, therefore, represent a novel potential oncogenic target for clinically approved Trk inhibitors in refractory PitNETs [57,58].

## Figures and Tables

**Table 1:** Individual patients’ (Pt) details, grouped according to PIT1, SF1, TPIT PitNET lineages, including the following: age at surgery; sex; positivity for prolactin (PRL), growth hormone (GH), thyroid stimulating hormone (TSH), follicle stimulating hormone (FSH), luteinizing hormone (LH) and adrenocorticotropic hormone (ACTH) immunostaining (IHC); Ki67% proliferation index (n/a, not available); functioning (F) or non-functioning (NF) clinical status; recurrent tumors (Rec) with associated aggressive (a) and metastatic (m) cases.

**PIT1 PitNETs**													
**INVASIVE (*n* = 11)**							**NON-INVASIVE (*n* = 13)**						
**Pt**	**Age**	**Sex**	**IHC**	**Clinical** **Status**	**Rec**	**Ki67** **(%)**	**Pt**	**Age**	**Sex**	**IHC**	**Clinical** **Status**	**Rec**	**Ki67** **(%)**
**1**	53	F	PRL	F	Y(a)	≥3	12 *	37	F	GH	F	N	≥3
**2**	19	M	GH	F	N	<3	**13**	52	F	GH	F	N	<3
**3**	16	M	PRL	NF	N	≥3	**14**	52	M	GH	F	N	<3
**4**	18	F	GH	NF	N	≥3	**15**	34	F	PRL	F	N	n/a
**5**	74	M	TSH	F	N	≥3	16 *	49	M	GH/PRL	F	N	<3
**6**	37	M	TSH	F	N	≥3	**17**	40	F	GH/PRL	F	N	≥3
**7**	25	F	GH	F	N	≥3	**18**	55	M	GH/PRL	F	N	≥3
**8**	21	M	PRL	F	N	n/a	**19**	36	F	PRL	F	N	n/a
**9**	76	F	GH	F	N	<3	**20**	26	M	PRL	F	N	n/a
**10**	14	M	GH/PRL	F	Y	≥3	21 *	50	M	GH	F	N	≥3
**11**	62	M	Pit1 only	NF	Y(m)	≥3	**22**	43	F	GH/PRL	NF	N	<3
							**23**	49	M	PRL	F	N	n/a
							**24**	32	F	GH	F	N	<3
**SF1 PitNETs**													
**INVASIVE (*n* = 12)**							**NON-INVASIVE (*n* = 12)**						
**Pt**	**Age**	**Sex**	**IHC**	**Clinical status**	**Rec**	**Ki67** **(%)**	**Pt**	**Age**	**Sex**	**IHC**	**Clinical status**	**Rec**	**Ki67** **(%)**
**25**	45	M	FSH/LH	NF	N	≥3	**37**	68	M	FSH/LH	NF	N	≥3
**26**	56	M	FSH/LH	NF	N	≥3	**38**	71	F	SF1 only	NF	Y	<3
**27**	73	F	FSH/LH	NF	N	≥3	39 *	71	M	FSH/LH	NF	N	<3
**28**	49	F	SF1 only	NF	N	≥3	**40**	67	M	SF1 only	NF	Y	≥3
**29**	55	F	SF1 only	NF	N	≥3	**41**	61	M	FSH/LH	NF	N	<3
**30**	48	M	FSH/LH	NF	N	≥3	**42**	46	M	FSH/LH	NF	N	≥3
**31**	53	M	FSH/LH	NF	N	≥3	**43**	75	M	FSH/LH	NF	N	<3
**32**	47	F	FSH/LH	NF	N	<3	**44**	74	M	SF1 only	NF	N	<3
**33**	69	M	FSH/LH	NF	Y	<3	**45**	66	M	FSH/LH	NF	N	<3
**34**	70	F	FSH/LH	NF	N	<3	**46**	39	M	FSH/LH	NF	N	<3
**35**	55	M	FSH/LH	NF	N	≥3	**47**	46	M	FSH/LH	NF	N	≥3
**36**	73	M	SF1 only	NF	N	≥3	**48**	69	F	FSH/LH	NF	N	<3
**TPIT PitNETs**													
**INVASIVE (*n* = 3)**							**NON-INVASIVE (*n* = 2)**						
**Pt**	**Age**	**Sex**	**IHC**	**Clinical status**	**Rec**	**Ki67** **(%)**	**Pt**	**Age**	**Sex**	**IHC**	**Clinical status**	**Rec**	**Ki67** **(%)**
**49**	57	M	ACTH	F	Y(a)	≥3	**52**	78	F	ACTH	F	N	<3
**50**	52	F	ACTH	NF	N	≥3	**53**	36	F	ACTH	F	N	≥3
**51**	26	F	ACTH	F	N	≥3							

* PitNETs that do not express TrkAIII mRNA.

**Table 2:** RT-PCR primers and conditions used in this study.

Target	Sequence	Denat	Ann	Ext	Amplicon
18S rRNA ****	F: 5′-AAACGGCTACCACATCCAAG-3′ R: 5′-CCTCGAAAGAGTCCTGTATTG-3′	30 s 94 °C	30 s 58 °C	30 s 72 °C	100 bp
TrkA ex 8-17 *	F: 5′-AACCCCTTCGGCCAGGCCTCC-3′ R: 5′-CTAGCCCAGGACATCCAGGTA-3′	1 m 94 °C	30 s 65 °C	1 m 72 °C	1298 bp TrkA
TrkA ex 1-8 *	F: 5′-ATGCTGCGAGGCGGACGGCGC-3′ R: 5′-GGAGGCCTGGCCGAAGGGGTT-3′	1 m 94 °C	30 s 68 °C	1 m 72 °C	1114 bp TrkA, 838 bp TrkAIII, 475 bp Δ2-7 TrkA
TrkA ex 5-8 *	F: 5′-AGAAGCTGCAGTGTCATGGG-3′ R: 5′-ATTGAGCACGGAGCCATTGA-3′	40 s 94 °C	30 s 58 °C	40 s 72 °C	452 bp TrkA 176 bp TrkAIII
SRSF2 ***	F: 5′-CTCCCGATGTGGAGGGTATG-3′ R: 5′-GAGATCGGCTGCGAGACC-3′	40 s 94 °C	30 s 58 °C	40 s 72 °C	408 bp
SF3B1 **	F: 5′-TGTGCATAAGATCCTCGTGGT-3′ R: 5′-ACACCATCTGTCCCACAACA-3′	40 s 94 °C	30 s 58 °C	4 s 72 °C	693 bp
SF3B1 (tDNA)	F: 5′-TAGGCTGCTGGTCTGGCTAC-3′ R: 5′-ATGGCACAGCCCATAAGAATAG-3′	30 s 95 °C	30 s 60 °C	1 m 72 °C	233 bp
U2AF1 **	F: 5′-CGGAGTATCTGGCCTCCATC-3′ R: 5′-GCAGCTCTCTGGAAATGGGCT-3′	40 s 94 °C	30 s 60 °C	40 s 72 °C	606 bp
HIF-1α **	F: 5′-TTCACCTGAGCCTAATAGTCC-3′ R: 5′-AAGTCTAAATCTGTGTCCTG-3′	30 s 94 °C	30 s 50 °C	30 s 72 °C	150 bp
HIF-2α ***	F: 5′-AGCCTCCATCTGCCATCAGTC-3′ R: 5′-CTTGCCATGCCTGACACCTTG-3′	30 s 94 °C	30 s 55 °C	30 s 72 °C	121 bp
JCPyV T-Ag *	F: 5′-ATATTATGACCCCCAAAACCATG-3′ R: 5′-GGTAGAAGACCCTAAGGACTTTCC-3′	40 s 94 °C	30 s 58 °C	40 s 68 °C	189 bp

RT-PCR: 40 cycles; * non-diluted cDNA (50 ng), ** 1:10 (5 ng); *** 1:100 (0.5 ng), and **** 1:1000 (0.05 ng) cDNA dilutions.

## Data Availability

The data presented in this study are available from the corresponding author upon reasonable request.
